# Identifying Blood Biomarkers and Physiological Processes That Distinguish Humans with Superior Performance under Psychological Stress

**DOI:** 10.1371/journal.pone.0008371

**Published:** 2009-12-18

**Authors:** Amanda M. Cooksey, Nausheen Momen, Russell Stocker, Shane C. Burgess

**Affiliations:** 1 Department of Basic Sciences, College of Veterinary Medicine, Mississippi State University, Mississippi State, Mississippi, United States of America; 2 Life Sciences and Biotechnology Institute, Mississippi State University, Mississippi State, Mississippi, United States of America; 3 Institute for Digital Biology, Mississippi State University, Mississippi State, Mississippi, United States of America; 4 Naval Aerospace Medical Research Laboratory, Pensacola, Florida, United States of America; 5 Navy Recruiting District, New England, Boston, Massachusetts, United States of America; 6 Department of Mathematics and Statistics, Mississippi State University, Mississippi State, Mississippi, United States of America; Charité-Universitätsmedizin Berlin, Germany

## Abstract

**Background:**

Attrition of students from aviation training is a serious financial and operational concern for the U.S. Navy. Each late stage navy aviator training failure costs the taxpayer over $1,000,000 and ultimately results in decreased operational readiness of the fleet. Currently, potential aviators are selected based on the Aviation Selection Test Battery (ASTB), which is a series of multiple-choice tests that evaluate basic and aviation-related knowledge and ability. However, the ASTB does not evaluate a person's response to stress. This is important because operating sophisticated aircraft demands exceptional performance and causes high psychological stress. Some people are more resistant to this type of stress, and consequently better able to cope with the demands of naval aviation, than others.

**Methodology/Principal Findings:**

Although many psychological studies have examined psychological stress resistance none have taken advantage of the human genome sequence. Here we use high-throughput -omic biology methods and a novel statistical data normalization method to identify plasma proteins associated with human performance under psychological stress. We identified proteins involved in four basic physiological processes: innate immunity, cardiac function, coagulation and plasma lipid physiology.

**Conclusions/Significance:**

The proteins identified here further elucidate the physiological response to psychological stress and suggest a hypothesis that stress-susceptible pilots may be more prone to shock. This work also provides potential biomarkers for screening humans for capability of superior performance under stress.

## Introduction

Attrition of trainees from the aviation program is a continuing concern for the U.S. Navy. Each late stage navy aviator training failure costs the taxpayer over $1,000,000 and ultimately results in decreased operational readiness of the fleet. Over the past 20 years the attrition rate of incoming aviation students has been between 15–25%. Failures occur for a variety of reasons including medical problems. However, most attritions result from academic or flight performance failures or requests to be dropped from the program (DoR; drop on request). Naval aviation is a highly stressful occupation requiring the ability to respond quickly and appropriately in dangerous situations. While there is no measure of the impact of psychological stress on attrition from the program, it makes a clear contribution to academic/flight performance failures and DoR. Biological screening of potential aviators based on performance under psychological stress could reduce all of the major contributing factors of attrition thus saving the Navy millions of dollars.

Potential aviators are currently selected using the Aviation Selection Test Battery (ASTB). The ASTB is a written test designed to evaluate math and verbal skills, mechanical comprehension, aviation and nautical information and spatial apperception. The ASTB has a strong predictive validity through primary flight training. While the ASTB evaluates many skills necessary to aviation, and it is correlated with performance, it does not account for the natural genetic variation in physiological stress response.

Once selected by the ASTB, all trainees undergo water survival training in the Modular Egress Training Simulator (METS) device (“helo-dunker”)—an underwater crash simulator that requires trainees to experience a water “crash” and perform an underwater egress while blindfolded. The helo-dunker is a highly demanding and stressful test.

Although human performance under psychological stress has been studied extensively, it has been primarily done psychometrically or using reductionist biological methods such as blood cortisol measurements [1]. Here we have used blood plasma of aviators undergoing METS training and a high-throughput proteomic approach to identify differential protein expression indicative of performance under psychological stress. Low and median- scoring performers (based on ASTB scores) differed from high-scoring performers in the regulation of four basic physiological processes: innate immunity, cardiac function, coagulation and plasma lipid physiology.

## Results and Discussion

### Protein Identification, Quantification, Normalization and Differentially Expressed Proteins

We identified between 2191–4526 proteins per sample triplicate. Data were normalized using an invariant set of proteins and compared to determine differential expression. Normalization of proteomics datasets is typically accomplished in one of two ways: 1) the addition of control proteins in known concentrations to the samples (a.k.a “spiking”) or 2) comparison of expression values to one or more pre-selected ‘housekeeping genes’. The problem with spiking is that it does not account for the differences in variation that are dependent on quantity of a protein in a sample and there is a 12-log dynamic range between the most and least common proteins in blood plasma [2]. Pre-selection of “housekeeping” proteins suffers from the same problems as spiking but also the assumption that the selected proteins do not change, may be wrong. However, what is clear (and logical) is that a significant proportion of proteins across the range of concentrations present in blood plasma will not change under specific conditions and that these can be used for accurate normalization that will take account of differences in variation that are dependent on quantity of a protein in a sample. Identification of invariant proteins *post hoc* guarantees normalization to data with unchanged expression. This concept has been applied to microarray transcriptome data [3] and proteome data [4]. Here we have used a method based on similar principles in which ΣXcorr was calculated for each protein and used to determine an invariant protein set *post hoc*. These proteins were used to normalize the ΣXcorr for the all the proteins in the dataset.

A set of 183 proteins were differentially expressed between subjects with high and low performance scores: 136 proteins were greater and 47 lower in the low scorers. Between high and median scorers 206 were differentially expressed: 155 greater and 51 lower among median scorers. A set of 331 proteins were differentially expressed between low and median vs. high scoring performers (Table S1).

### Gene Ontology

**Figure 1:**
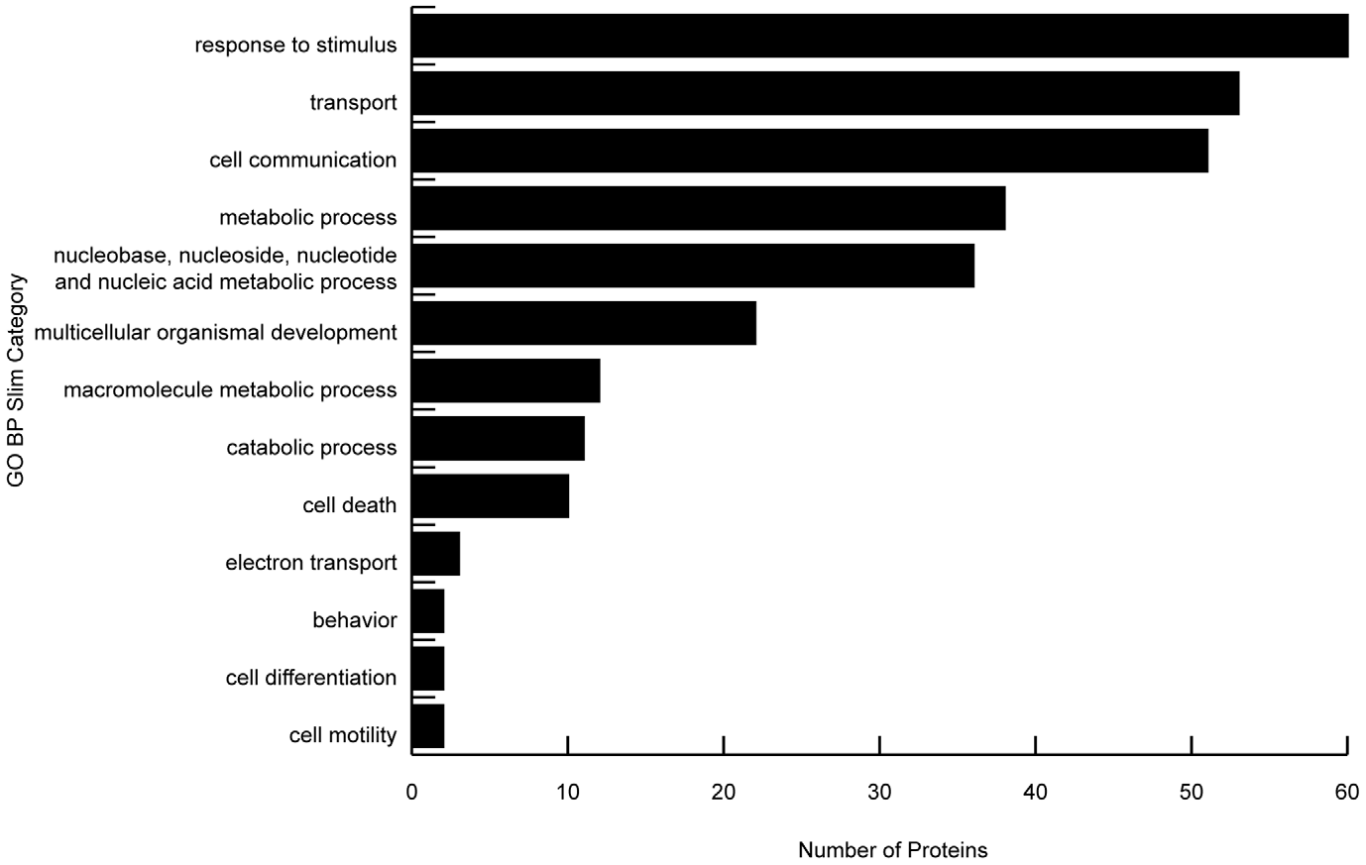
GO slim of differentially expressed proteins. All 183 differentially expressed proteins between high scoring and low scoring performers were GO annotated and slimmed according to the GOA GO slim. This graph shows the number of proteins annotated to each slim category.

We next obtained up-to-date (23JUL08) GO annotations for, and then modeled based on GO biological process (GOBP), these differentially expressed proteins. The Gene Ontology Annotation (GOA) slim for biological process was applied to the differentially expressed proteins (Figure 1). More proteins had GOBP “response to stimulus” than any other GO slim category. Response to stimulus is defined as: “a change in state or activity of a cell or an organism (in terms of movement, secretion, enzyme production, gene expression, etc.) as a result of a stimulus”. This category includes responses to a variety of stimuli including stress. The use of GO slims allows for the identification of general physiological processes important to performance under stress. However, these categories are very broad. Figure S1 shows the specific annotations identified as a part of the response to stimulus GO slim category. Differentially regulated proteins were annotated to each of the shaded terms. Not surprisingly, most (13/18) of these terms are a part of immune response and response to stress.

### Ingenuity Pathways Analysis

**Figure 2:**
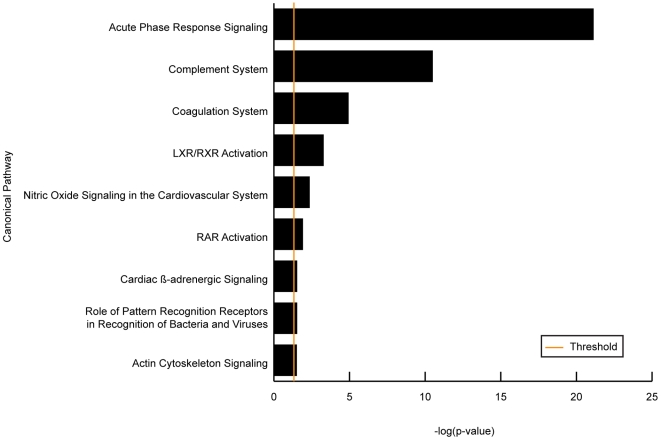
Pathways significantly affected by performance under psychological stress. Differentially expressed proteins were mapped to canonical pathways using Ingenuity Pathways Analysis software. Nine pathways were found to be significantly (Fisher's Exact; p<0.05) represented by the differentially expressed proteins. The threshold is the −log of the p-value (0.05).

To better understand the physiological responses related to performance under stress we modeled our biomarkers using Ingenuity Pathways Analysis software (IPA). Because our aim was to identify the protein expression that distinguishes stress-resistant high-scoring performers from their stress-susceptible low performing colleagues, we compared expression levels of low and median-scoring vs. high-scoring performers. This resulted in nine canonical pathways (Figure 2): acute phase response (APR), complement system, coagulation system, LXR/RXR activation, nitric oxide signaling in the cardiovascular system, cardiac b-adrenergic signaling, role of pattern recognition receptors in the recognition of bacteria and viruses, RAR activation and actin cytoskeleton signaling. Together these pathways represent 4 basic physiological processes: innate immunity, coagulation, cardiac function and plasma lipid regulation. Each of these processes is discussed in detail.

### Innate Immunity

Several innate immune response pathways were differentially expressed in low and median scoring performers compared to high-scoring performers: acute phase response, complement system (Figure S2), role of pattern recognition in recognition of bacteria and viruses and RAR activation.

Acute phase response is an evolutionarily conserved systemic physiological response to infection, injury or stress resulting in the increased or decreased plasma concentration of several proteins called acute phase proteins (APP). In addition to APP, we also found up-regulation of three regulatory members of the APR pathway among low and median-scoring performers. We also found a greater increase in plasma IL-1, PI3K and NFkB among low and median- scoring performers. These three regulatory proteins are necessary for the activation of the acute phase response. We identified 22 APP that were up-regulated among poor and median performers in response to psychological stress.

Several complement system proteins were differentially expressed (Figure S2). As with acute phase response, the complement system is a potent activator of inflammation that has been previously associated with both chronic [5] and acute psychological stress [6]. Proteins of the complement system act as a cascade ultimately initiating inflammation and formation of the membrane attack complex, responsible for pathogen cytolysis. We identified many proteins belonging to the classical and alternative pathways of the complement cascade for which plasma concentrations are inversely correlated with performance under psychological stress. Low and median scoring performers showed greater increases in plasma concentrations of these proteins than did high-scoring performers. Identification of these proteins supports our above finding that poor performance under psychological stress is correlated with a pro-inflammatory state. Indeed, this is in agreement with previous findings that several complement proteins were elevated before and during both lab and naturalistic psychological stress [7].

We also found up-regulation of the RELA and PIK3 proteins present in the pattern recognition and RAR activation pathways. Differential expression of these proteins also suggests that poor and median-scoring performers experience a greater pro-inflammatory response to psychological stress. From an evolutionary perspective, inflammation as a part of the fight, flight or freeze response would prepare organisms for infections resulting from injury. Accordingly, individuals who performed poorly in a stressful situation would be more likely to incur injury and require an immune response.

### Coagulation

We found several members of the intrinsic coagulation pathway to be increased in low and median-scoring performers compared to high-scoring performers (Figure S3). Specifically, low and median-scoring performers show increases in both the coagulation and fibrinolysis pathways; overall tending toward fibrinolysis compared to their high-scoring counterparts. In support of our findings, acute psychological stress causes up-regulation of both the coagulant and fibrinolysis pathways, resulting in overall hyper-coagulability [8]. More significantly, a pro-coagulant response has been directly correlated with anticipatory appraisal of a stressor [9].

Simultaneous upregulation of both coagulation and fibrinolysis is continuous and serves to maintain hemostasis. As coagulatory response to stress increases, greater compensatory mechanisms are necessary to maintain the balance between coagulation and fibrinolysis. Greater increases among poor and median-scoring performers, compared to high-scoring performers, suggest increased responsiveness among poor and median-scoring performers resulting in greater shifts in this fine balance. A tendency toward fibrinolysis is a compensatory response to prevent disseminated intravascular coagulation (DIC) which can result from shock. DIC is elicited by inflammatory cytokines also indicative of the greater innate immune response discussed above. However, paradoxically, an excessive anticoagulant response can itself induce DIC and thus shock [10] and we hypothesize that the low and moderate performers may be more prone to shock.

### Cardiac Function

Three pathways affecting cardiac function were differentially regulated in poor and median performers compared to superior performers. The cardiac b-andrenergic signaling, nitric oxide signaling in the cardiovascular system and actin cytoskeleton signaling pathways were more greatly up-regulated among poor and median-scoring performers compared to high-scoring performers.

Beta-adrenergic signaling in the cardiac system is elicited by the release of epinerphrine (adrenaline) in response to stress and is responsible for the increased heart rate, contractility and vasodilation central to a fight-or-flight response. Nitric oxide produced within cardiac muscle cells enhances the effect of catecholamines (eg. epinephrine) either by increasing the release or preventing the reuptake of catecholamines at a pre-synaptic level [11], [12]. Among the proteins identified in the actin cytoskeleton signaling pathway was F-actin (filamentous actin), the major component of muscle. Although F-actin is found in all muscle and could represent changes taking place throughout the body, in light of the other pathways significantly affected here it is likely that cardiac muscle is most affected. Increased F-actin production likely results from the increased heart rate and contractility discussed above. More F-actin would be necessary for both addition to and repair of the muscle tissue. Taken together, greater up-regulation of these pathways among poor and median-scoring performers suggests an enhanced cardiac epinephrine response to psychological stress.

### Plasma Lipid Physiology

Previously, increased plasma cholesterol has been linked to stress [13]. Here, we found several proteins of the LXR/RXR activation pathway were more greatly increased among poor and median-scoring performers compared to high-scoring performers: APOA1, APOC1, APOC4, RBP4, AGT, HPX, CP and ITIH4. Up-regulation of the proteins found here would be expected to cause both increased cholesterol efflux and reduced plasma lipid clearance resulting in increased plasma lipid levels. APOA1, APOC1 and APOC4 are exchangeable apolipoproteins involved in cholesterol efflux [14], a critical step in reverse cholesterol transport (RCT), the process by which cholesterol is removed from the peripheral tissues and returned to the liver. Exchangeable apolipoproteins bind effluxed cholesterol in the blood stream. Increases in plasma apolipoprotein concentrations promote cholesterol efflux from the peripheral tissues [14]–[16]. Plasma RBP4 concentration is directly correlated with triacylglycerol [17]–[20], serum total cholesterol [21], [22] and LDL [22], [23] concentrations. ITIH4 has been correlated with hypercholesterolemia [24].

Poor and median-scoring performers would also experience reduced lipid clearance. APOC1 reduces lipid clearance through inhibition of APOE-mediated hepatic clearance via the low-density lipoprotein receptor (LDLR) [25], very low-density lipoprotein receptor (VLDLR) and the alternate clearance pathway via binding of LDLR-related protein (LRP) [26] and inhibition of lipoprotein lipase (LPL)-mediated triglyceride lipolysis [27]. Increased APOC1 has been associated with elevated plasma VLDL, triglyceride and free fatty acid levels [28]. A2M decreases the uptake of LDL cholesteryl ester via APOE [29]. These findings, taken together, suggest an increase in plasma lipids among low and median-scoring performers in response to psychological stress.

### Conclusion

We have used a novel statistical method to ensure accurate quantitative comparisons between subjects with various performance levels under psychological stress. This invariant set method does not rely on the assumption that ‘housekeeping genes’ have unchanged expression. Normalization to several proteins across a range of expression levels ensures appropriate normalization of all sample proteins. We have identified 331 proteins differentially expressed in poor and median-scoring performers compared to high-scoring performers under psychological stress. Four basic physiological processes were related to inferior performance: innate immunity, coagulation, cardiac function and plasma lipid physiology. Poor and median-scoring performers experienced more inflammation, coagulation and fibrinolysis, cardiac response to epinephrine and increased plasma lipid concentrations than did their superior-scoring counterparts. These proteins and processes provide value for developing a complementary tool for selecting not only Naval aviators but also trainees in other fields in which superior performance under extreme psychological stress is required. Regardless of the underlying physiology, these proteins in themselves provide a foundation for developing the first robust blood biomarker for rapidly quantifying human stress responses.

## Materials and Methods

### Ethics Statement

All sample collection took place at the Water Survival Training facility at Naval Air Station Pensacola and was approved by the Naval Aerospace Medical Research Laboratory Institutional Review Board (human use research protocol number NAMRL.2005.0003). Written informed consent was obtained from all participants.

### Subjects and Sample Collection

Trainees were required to successfully egress the METS (a simulated helicopter fuselage) three times in six attempts. Blood samples were collected from 22 trainees before (pre-stress) and after (post-stress) their METS training. Pre-stress samples were collected 24 h prior to the METS and post-stress samples were collected no more than 20 minutes following the last successful egress.

Plasma samples were collected and stored according to the conventional method. Blood was collected into EDTA vaccutainers, centrifuged (900 x g, 15 min, room temperature) to collect plasma and then stored in a plain vaccutainer. After collection, each sample was aliquoted into 200 µl aliquots and frozen at −80°C. The pre- and post-stress plasma samples were labeled accordingly with subject numbers in order to match the data to each of the participants. All the plasma samples were sent to Mississippi State University for analysis.

The Aviation Selection Test Battery (ASTB) is the only test used by the Navy, Marine Corp and Coast guard for aviation program selection. It consists of 4 subtests: Math and Verbal Test, Mechanical Comprehension Test, Aviation and Nautical Information Test, and Spatial Apperception Test. The ASTB subtests are weighted and the following composite scores are computed: 1) Academic Qualification Rating (AQR); 2) Pilot Flight Aptitude Rating (PFAR); 3) Flight Officer Flight Aptitude Rating and 4) Officer Aptitude Rating. AQR and PFAR scores are used for pilot programs [30]. Composite scores were collected for all subjects. Because we were interested in pilot performance AQR+PFAR was used as a measure of ASTB score.

### Experimental Design

Of the 22 students 9 (all male) were chosen, based on their ASTB scores, for further analysis: 3 students with the highest scores (high performance group), 3 with the lowest scores (low performance group) and 3 with median scores (median performance group).

### Abundant Protein Depletion

In order to increase proteome coverage, six of the most abundant plasma proteins (albumin, IgG, IgA, anti-trypsin, transferrin and haptoglobin) were depleted using the Agilent Multiple Affinity Removal System, 4.6×50 mm LC column (Agilent Technologies). Plasma from each sample was diluted 1∶5 in Buffer A. Particulates were removed using 0.22 µm spin filters; 1 min at 16 000 x g. Filtered plasma samples were then loaded into the Thermo-Separation Products AS3000 autosampler attached to the Agilent 1100 LC system equipped with a quaternary pump and diode array detector. Samples were run according to the LC timetable indicated by the column manufacturer and collected using the Gilson FC203B fraction collector. Samples in both the autosampler and fraction collector were maintained at 4°C throughout the LC protocol. Samples were collected between 1.7–4.5 min of the 20 min protocol ensuring collection of the high-abundant protein peak as detected by UV. Following abundant protein depletion, sample concentration was determined using the 2-D Quant Kit (Amersham Biosciences) and found to have been reduced by ∼90% as predicted by the column manufacturer. Each sample was divided into three 100 µg technical replicates. Each replicate was frozen (−80°C, 15 min) and lyophilized (FreeZone 2.5 L Benchtop freeze dry system, Labconco Corporation, Kansas City, MO. USA; 2 hr).

### Proteomics

Samples were diluted in ammonium bicarbonate (0.1 M; 100 µl), reduced using dithiothreitol (5 mM; 5 min; 65°C), alkylated using iodoacetamide (10 mM; 30 min; 30°C) and trypsin-digested (molecular biology grade trypsin; Promega Corporation, Madison, WI; 50∶1 protein∶trypsin [w/w]; 16 h; 37°C). Peptides were desalted using a peptide macrotrap (Michrom Bioresources, Inc., Auburn, CA, USA) and eluted in a 95% ACN, 0.01% TFA solution. Desalted peptides were dried in a vacuum centrifuge (ThermoElectron) and stored (−80°C) for further analysis.

Dried samples were resuspended in 20 µl of 0.1% formic acid, 5% acetonitrile. LC analysis was accomplished by SCX followed by RP LC coupled directly in line with ESI IT MS. Samples were loaded into a LC gradient ion exchange system containing a Thermo Separations P4000 quaternary gradient pump (ThermoElectron) coupled with a 0.32×100 mm BioBasic SCX column. A flow rate of 3 µL/min was used for both SCX and RP columns. A salt gradient was applied in steps of 0, 10, 15, 20, 25, 30, 35, 40, 45, 50, 57, 64, 90 and 700 mM ammonium acetate in 5% ACN, 0.1% formic acid and the resultant peptides loaded directly into the sample loop of a 0.18×100 mm BioBasic C18 RP LC column of a ProteomeX workstation (Thermo Electron). The RP gradient used 0.1% formic acid in ACN and increased the ACN concentration in a linear gradient from 5% to 30% in 30 min and then 30% to 65% in 9 min followed by 95% for 5 min and 5% for 15 min. The spectrum collection time was 59 min for every SCX step. The Deca LCQ IT mass spectrometer was configured to optimize the duty cycle length with the quality of data acquired by alternating between a single full MS scan followed by three tandem MS scans on the three most intense precursor masses (as determined by Xcalibur mass spectrometer software in real time) from the full scan. The collision energy was normalized to 35%. Dynamic mass exclusion windows were set at 2 min and all of the spectra were measured with an overall mass/charge (*m*/*z*) ratio range of 300–1700 Th.

Tandem mass spectra were used to search a database of all human RefSeq proteins downloaded directly from the National Center for Biotechnology Institute (NCBI; 27MAR07) and a decoy database using TurboSEQUEST (Bioworks Browser 3.2; ThermoElectron). Trypsin digestion was applied *in silico* to the modified database including mass changes due to cysteine carbamidomethylation and methionine oxidation. The peptide (MS precursor ion) mass tolerance was set to 1.5 Da and the fragment ion (MS2) mass tolerance was set to 1.0 Da. For both the real and decoy databases, peptides were filtered by Xcorr >1.0 and exported for further analysis. Real database identifications were determined by comparison of the real and decoy database matches using our *DecoyPepFilter* program. Peptides were grouped by charge state (+1, +2, +3) and sorted by Xcorr x ΔCn. For each real database peptide match within a charge state group, the percentage of decoy database matches with the same or higher Xcorr x ΔCn was calculated. Peptides were considered real if ≤1% of decoy database matches had the same or higher Xcorr x ΔCn. Protein identifications have been submitted to the proteomics identifications database (PRIDE, [31], [32]), accession numbers 10075–10092 inclusive.

### Data Normalization and Calculation of Differential Expression

Sample normalization was done based on the principle that many proteins would not vary between samples and these proteins can be used as internal standards [3], [4]. To identify these proteins and then use them for normalization we did global lowess normalization and constructed an invariant protein set. For the jth sample denote the ΣXCorr associated with protein k as Y*_kj_* where *k* = 1,…,*m* and *j* = 1,…, *n*. A median mock sample was constructed with *jth* element, Y*_j0_*  =  median [Y*_j_*_1_,…, Y*_jn_*]. Within each sample the Y*_kj_*s were ranked. We denote the rank of protein *k* within sample *j* as *R_kj_*. A rank sum of squares (*RSS*) was calculated for each kth protein as *RSS_k_*  =  *P^n^_i_* = 1(*R_ki_ −R_k0_*)^2^. We plotted log(*RSS_k_*) versus log(*R_k_*_0_). The invariant protein set was determined by visual inspection of the plot. Invariant proteins were chosen so they covered the full range of Y*_kj_* scores. A lowess normalization was then performed by fitting a lowess smooth [33] to the Y*_kj_* scores of the invariant protein set. Once all ΣXcorr values were normalized between samples we then calculated the differential expression for each subject using post-stress ΣXcorr – control ΣXcorr  =  ΔΣXcorr. ΔΣXcorr values were then compared between subjects grouped by ASTB scores (e.g. high performance group vs. low performance group). ΔΣXcorr values were subjected to monte carlo resampling with replacement (1000 iterations) to generate a p-value exactly as described previously [34]. Differential expression was considered statistically significant when p<0.05.

### Gene Ontology Annotation

Gene Ontology annotations for all differentially expressed proteins were obtained using *GORetriever* [35]. These annotations were grouped into broader categories using *GOSlimViewer* [35] and the GOA slim.

### Canonical Genetic Network and Pathway Modeling

Data were analyzed using Ingenuity Pathways Analysis (Ingenuity®Systems). Canonical pathways analysis identified the pathways from the Ingenuity Pathways Analysis library of canonical pathways that were most significant to the data set. Genes from the data set that were associated with a canonical pathway in the Ingenuity Pathways Knowledge Base were considered for the analysis. A Fisher's Exact test was used to calculate a p-value determining the probability that the association between the genes in the dataset and the canonical pathway is explained by chance alone. Pathways were considered to be statistically significantly associated with the dataset if p<0.05.

## Supporting Information

Figure S1 Response to stimulus annotations. Proteins annotated to the response to stimulus slim category were mapped to their most specific GO terms. Terms to which identified proteins were annotated are shaded.
(0.34 MB JPG)

Figure S2 Coagulation Sytstem. The intrinsic pathway of coagulation was differentially regulated according to performance under psychological stress. Proteins we identified within this pathway are in color. Proteins up-regulated in low and median scoring performers compared to high scoring performers are red.
(0.37 MB JPG)

Figure S3 Complement System. The complement system pathway was differentially regulated according to performance under psychological stress. Proteins we identified within this pathway are in color. Proteins up-regulated in low and median scoring performers compared to high scoring performers are red.
(0.31 MB JPG)

Table S1 Differentially Expressed Proteins
(0.06 MB PDF)
