# Supplementary figures and images for: Identifying Blood Biomarkers and Physiological Processes That Distinguish Humans with Superior Performance under Psychological Stress
Source: PLoS One. 2009 Dec 18;4(12):e8371. doi: 10.1371/journal.pone.0008371 (PMC2791215; doi:10.1371/journal.pone.0008371)

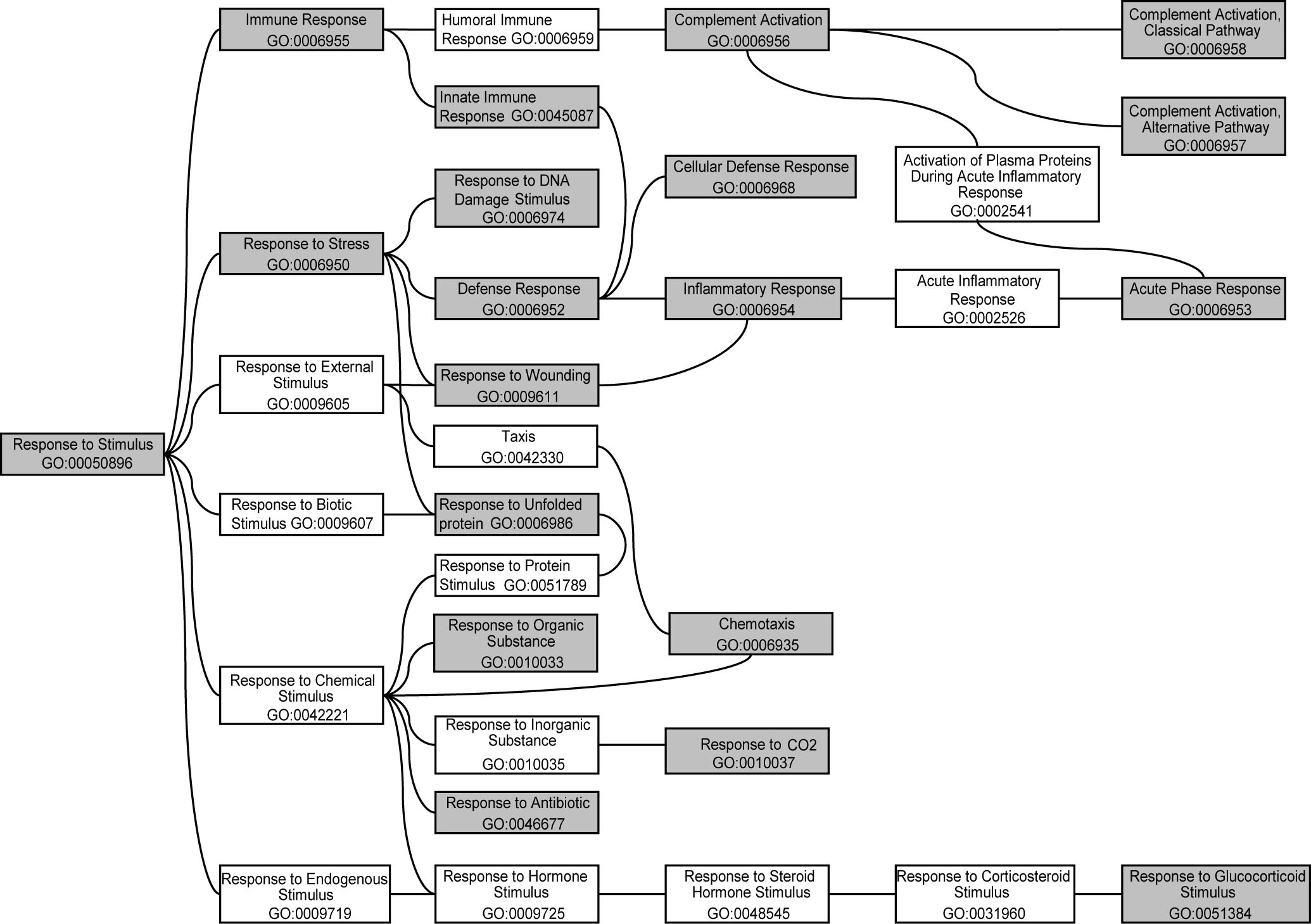

Supplement: Figure S1 — Response to stimulus annotations. Proteins annotated to the response to stimulus slim category were mapped to their most specific GO terms. Terms to which identified proteins were annotated are shaded. (0.34 MB JPG) [file pone.0008371.s001.jpg]

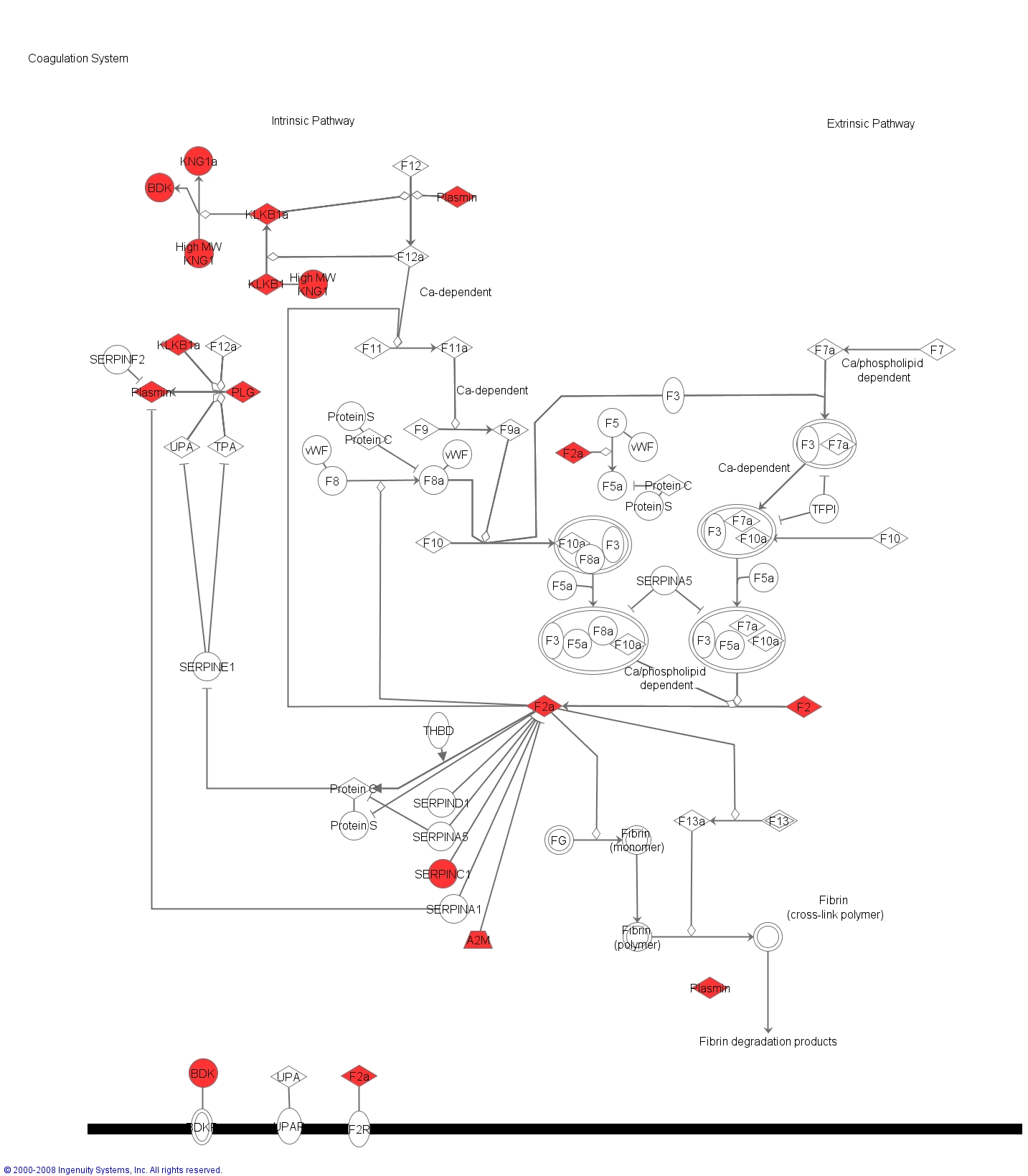

Supplement: Figure S2 — Coagulation Sytstem. The intrinsic pathway of coagulation was differentially regulated according to performance under psychological stress. Proteins we identified within this pathway are in color. Proteins up-regulated in low and median scoring performers compared to high scoring performers are red. (0.37 MB JPG) [file pone.0008371.s002.jpg]

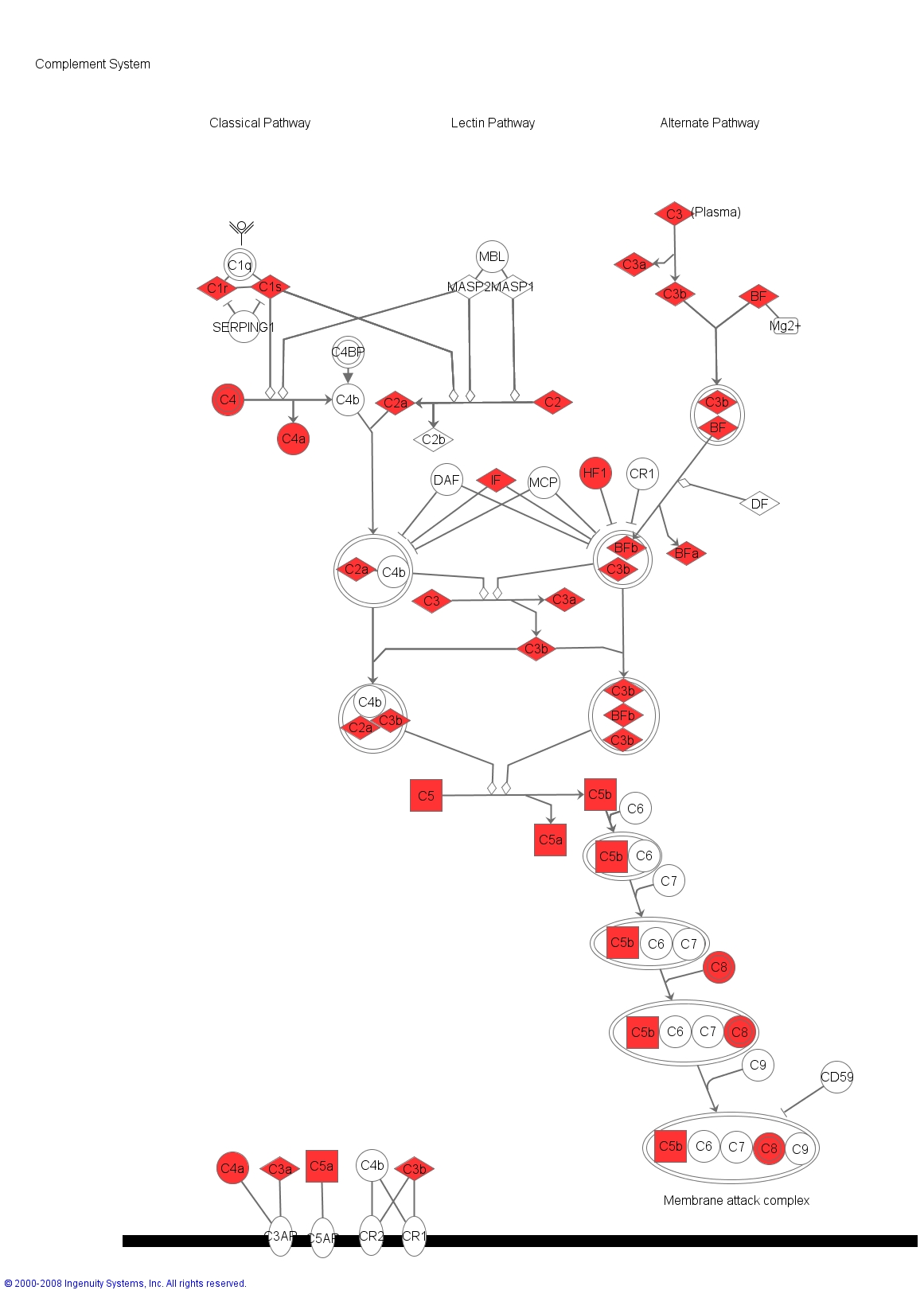

Supplement: Figure S3 — Complement System. The complement system pathway was differentially regulated according to performance under psychological stress. Proteins we identified within this pathway are in color. Proteins up-regulated in low and median scoring performers compared to high scoring performers are red. (0.31 MB JPG) [file pone.0008371.s003.jpg]
