# Supplementary material for: Identifying Blood Biomarkers and Physiological Processes That Distinguish Humans with Superior Performance under Psychological Stress
Source: PLoS One. 2009 Dec 18;4(12):e8371. doi: 10.1371/journal.pone.0008371 (PMC2791215; doi:10.1371/journal.pone.0008371)
Supplement: Table S1 — Differentially Expressed Proteins (0.06 MB PDF) [file pone.0008371.s004.pdf]

Table S1. Differentially Expressed Proteins

Proteins Up-regulated in Low-Scorers Compared to High-Scorers

Genbank Accession	Protein Name
NP_001605	actin, gamma 1 propeptide
NP_001636	apolipoprotein C-I
NP_000479	serpin peptidase inhibitor, clade C, member 1
NP_001228	cyclin A
NP_000496	coagulation factor XII
NP_002206	inter-alpha (globulin) inhibitor H1
NP_002903	DNA polymerase zeta
NP_002968	sodium channel, voltage-gated, type IX, alpha
NP_003183	beta-tubulin cofactor C
NP_000033	apolipoprotein H
NP_000046	butyrylcholinesterase
NP_000087	ceruloplasmin
NP_000056	complement component 6
NP_004920	calbindin 1
NP_004961	insulin-like growth factor binding protein, acid labile subunit isoform 2
NP_005413	tectorin alpha
NP_006854	leukocyte immunoglobulin-like receptor, subfamily A (with TM domain), member 1
NP_055488	tetratricopeptide repeat domain 35
NP_055710	KIAA1009 protein
NP_060570	EF-hand domain (C-terminal) containing 1
NP_065169	sal-like 4
NP_000604	hemopexin
NP_002454	myxovirus resistance protein 2
NP_079221	RMI1, RecQ mediated genome instability 1, homolog
NP_079222	hypothetical protein LOC80011
NP_116194	zinc finger and SCAN domain containing 10
NP_000054	complement component 2 isoform 1
NP_001924	dihydrolipoamide S-succinyltransferase (E2 component of 2-oxo-glutarate complex)
NP_055253	interleukin 1 family, member 8 isoform 1
NP_620135	synaptotagmin-like 5 isoform 1
NP_663318	apolipoprotein L1 isoform b
NP_061974	apolipoprotein M
NP_690595	RNA binding motif protein 10 isoform 2
NP_059136	mouse Mdm1 nuclear protein homolog isoform 1
NP_002882	Ras protein-specific guanine nucleotide-releasing factor 1 isoform 1
NP_116168	coiled-coil domain containing 142
NP_116107	major facilitator superfamily domain containing 9
NP_060808	WD repeat domain 52 isoform 2
NP_066267	ankyrin 3 isoform 1
NP_004136	myosin IXB isoform 1
NP_009086	zona pellucida glycoprotein 3 isoform 2
NP_958850	complement component 1, s subcomponent
NP_976053	hypothetical protein LOC399670
NP_055790	microtubule associated serine/threonine kinase 1
NP_060020	mesoderm induction early response 1, family member 2
NP_068810	v-rel reticuloendotheliosis viral oncogene homolog A isoform 1
NP_703144	killer cell immunoglobulin-like receptor, three domains, long cytoplasmic tail, 3
NP_079146	DEP domain containing 2 isoform a
NP_778228	pleckstrin homology domain containing, family A member 7
NP_997544	zinc finger CCCH-type containing 14 isoform 3
NP_955384	putative UST1-like organic anion transporter
NP_060024	poly (ADP-ribose) polymerase family, member 14
NP_001004060	nodal modulator 2 isoform 1
NP_001862	pancreatic carboxypeptidase B1
NP_001007526	NACHT and WD repeat domain containing 1

NP_001011553	cell division cycle 10 isoform 2
NP_000005	alpha-2-macroglobulin
NP_000664	class IV alcohol dehydrogenase, mu or sigma subunit
NP_000473	apolipoprotein A-IV
NP_001028725	spectrin domain with coiled-coils 1 NSP5b3b
NP_001030598	general transcription factor IIIC, polypeptide 2, beta 110kDa
NP_006022	pericentrin
NP_659473	coiled-coil domain containing 46 isoform a
NP_056988	eukaryotic translation initiation factor 5B
NP_060244	CDK5 regulatory subunit associated protein 1-like 1
NP_002967	sodium channel, voltage-gated, type VII, alpha
NP_000795	folate receptor 3
XP_001132268	PREDICTED: hypothetical protein
XP_001128594	PREDICTED: hypothetical protein
XP_001126166	PREDICTED: similar to MUC19
XP_062871	PREDICTED: similar to Temporarily Assigned Gene name family member (tag-58)
XP_001129821	PREDICTED: hypothetical protein
XP_001132605	PREDICTED: hypothetical protein
NP_079052	NKF3 kinase family member
XP_001129553	PREDICTED: hypothetical protein
XP_001129374	PREDICTED: hypothetical protein
NP_001846	alpha 1 type XV collagen
NP_207837	cell recognition protein CASPR4 isoform 1
NP_113665	yippee-like 3 isoform 1
NP_689472	leucine-rich repeat kinase 1
NP_114141	hemicentin 1
NP_057232	SH3 and multiple ankyrin repeat domains 1
NP_940864	doublecortin domain containing 5
NP_001073867	KIAA0368 protein
NP_001074008	coiled-coil domain containing 150
NP_003913	hect domain and RCC1-like domain 1
NP_997246	Nck-associated protein 5 isoform 1
NP_001124	afamin
NP_001869	cellular retinoic acid binding protein 2
NP_000497	coagulation factor II
NP_000403	histidine-rich glycoprotein
NP_000599	orosomucoid 2
NP_000292	plasminogen
NP_000030	apolipoprotein A-I
NP_006813	RAB40B, member RAS oncogene family
NP_055722	FERM and PDZ domain containing 1
NP_057257	HemK methyltransferase family member 1
NP_064528	partner of NOB1
NP_079182	stearoyl-CoA desaturase 5 isoform b
NP_036290	F-box and leucine-rich repeat protein 3
NP_570602	alpha 1B-glycoprotein
NP_009060	zinc finger protein of the cerebellum 2
NP_689865	zinc finger protein 48
NP_002462	myosin heavy chain 6
NP_002209	inter-alpha (globulin) inhibitor H4
NP_000574	vitamin D-binding protein
NP_009201	FK506 binding protein 9
NP_061875	ETAA16 protein
NP_001726	complement component 5
NP_937895	gelsolin isoform b
NP_942559	prickle-like 2
NP_997647	fibronectin 1 isoform 1
NP_476508	alpha 3 type VI collagen isoform 5

NP_006735	retinol-binding protein 4, plasma
NP_006581	ubiquitin specific protease 39
NP_009224	complement component 4A
NP_001701	complement factor B
NP_068838	zinc finger protein 711
NP_002207	inter-alpha globulin inhibitor H2 polypeptide
NP_065869	hypothetical protein LOC57578
NP_000883	plasma kallikrein B1
NP_055836	zinc finger protein 292
XP_950677	PREDICTED: hypothetical protein
NP_150229	ATP-binding cassette protein C12
NP_002152	isoleucine tRNA synthetase
NP_004992	NADH dehydrogenase (ubiquinone) 1 alpha subcomplex, 7, 14.5kDa
NP_000375	apolipoprotein B
NP_006505	sodium channel, voltage-gated, type X, alpha
NP_001026	cardiac muscle ryanodine receptor
NP_055646	lupus brain antigen 1
XP_001128246	PREDICTED: hypothetical protein
XP_942645	PREDICTED: similar to Temporarily Assigned Gene name family member (tag-58)
NP_056936	zinc finger protein 117
NP_000055	complement component 3
NP_536350	GNAS complex locus XLas
NP_001073347	cyclic nucleotide gated channel alpha 3 isoform 2

Proteins Down-Regulated in Low-Scorers Compared to High-Scorers

**Genbank Accession**	**Protein Name**
NP_003562	phakinin
NP_005856	protease, serine, 16
NP_036553	rearranged L-myc fusion
NP_055766	zinc finger protein 365 isoform A
NP_077015	caspase recruitment domain protein 14 isoform 1
NP_114432	BRCA1 interacting protein C-terminal helicase 1
NP_647538	attractin isoform 2
NP_659434	folliculin isoform 1
NP_689426	p53 target zinc finger protein isoform 2
NP_703149	extraembryonic, spermatogenesis, homeobox 1-like
NP_705835	luman-recruiting factor
NP_055904	p53-associated parkin-like cytoplasmic protein
NP_751950	interleukin 15 receptor, alpha isoform 2
NP_003598	CDC42-binding protein kinase alpha isoform B
NP_003713	tumor protein p63 isoform 1
NP_938204	c-Maf-inducing protein isoform C-mip
NP_940818	chromatin modifying protein 2A
NP_942596	zinc finger protein 160
NP_945346	hypothetical protein LOC374864 isoform 2
NP_055548	zinc finger, FYVE domain containing 16
NP_694574	Nedd4 binding protein 1
NP_006827	GCN1 general control of amino-acid synthesis 1-like 1
NP_001973	erbB-3 isoform 1
NP_001011649	CDK5 regulatory subunit associated protein 2 isoform b
NP_055516	SECIS binding protein 2-like
NP_006488	hypermethylated in cancer 1 isoform 1
NP_067072	sorting nexin 6 isoform a
XP_935700	PREDICTED: hypothetical protein
XP_949623	PREDICTED: hypothetical protein
NP_079421	hypothetical protein LOC80217
XP_001127275	PREDICTED: immunoresponsive 1 homolog (mouse)
NP_055885	zinc finger CCCH-type containing 13

NP_006693	patatin-like phospholipase domain containing 6
NP_689594	xin actin-binding repeat containing 2 isoform 1
NP_055845	furry-like
NP_001638	apolipoprotein D
NP_002590	phosphodiesterase 2A isoform 1
NP_002821	protein tyrosine phosphatase, non-receptor type 4
NP_002890	retinol binding protein 1, cellular isoform a
NP_113628	transmembrane protein 7
NP_002060	guanine nucleotide binding protein (G protein), alpha inhibiting activity polypeptide 1
NP_940959	RNA binding motif protein 43
NP_055073	synaptonemal complex protein 2
NP_005838	solute carrier family 23 (nucleobase transporters), member 1 isoform a
NP_005086	zinc finger protein 262
NP_000414	keratin 2
NP_001001894	tetratricopeptide repeat domain 3

Proteins Up-Regulated in Median-Scorers Compared to High Scorers

Genbank Accession	Protein Name
NP_001124	afamin
NP_001638	apolipoprotein D
NP_001869	cellular retinoic acid binding protein 2
NP_000497	coagulation factor II
NP_000403	histidine-rich glycoprotein
NP_000599	orosomucoid 2
NP_000292	plasminogen
NP_000030	apolipoprotein A-I
NP_006813	RAB40B, member RAS oncogene family
NP_055722	FERM and PDZ domain containing 1
NP_057257	HemK methyltransferase family member 1
NP_064528	partner of NOB1
NP_079182	stearoyl-CoA desaturase 5 isoform b
NP_036290	F-box and leucine-rich repeat protein 3
NP_570602	alpha 1B-glycoprotein
NP_009060	zinc finger protein of the cerebellum 2
NP_689865	zinc finger protein 48
NP_002462	myosin heavy chain 6
NP_002209	inter-alpha (globulin) inhibitor H4
NP_000574	vitamin D-binding protein
NP_009201	FK506 binding protein 9
NP_061875	ETAA16 protein
NP_001726	complement component 5
NP_937895	gelsolin isoform b
NP_942559	prickle-like 2
NP_997647	fibronectin 1 isoform 1
NP_476508	alpha 3 type VI collagen isoform 5
NP_006735	retinol-binding protein 4, plasma
NP_009224	complement component 4A
NP_001701	complement factor B
NP_068838	zinc finger protein 711
NP_002207	inter-alpha globulin inhibitor H2 polypeptide
NP_065869	hypothetical protein LOC57578
NP_000883	plasma kallikrein B1
NP_055836	zinc finger protein 292
XP_950677	PREDICTED: hypothetical protein
NP_150229	ATP-binding cassette protein C12
NP_002152	isoleucine tRNA synthetase
NP_004992	NADH dehydrogenase (ubiquinone) 1 alpha subcomplex, 7, 14.5kDa
NP_000375	apolipoprotein B

NP_006505	sodium channel, voltage-gated, type X, alpha
NP_001026	cardiac muscle ryanodine receptor
NP_055646	lupus brain antigen 1
XP_001128246	PREDICTED: hypothetical protein
XP_942645	PREDICTED: similar to Temporarily Assigned Gene name family member (tag-58)
NP_056936	zinc finger protein 117
NP_000055	complement component 3
NP_536350	GNAS complex locus XLas
NP_001073347	cyclic nucleotide gated channel alpha 3 isoform 2
NP_001609	poly (ADP-ribose) polymerase family, member 1
NP_001366	deoxyribonuclease II, lysosomal
NP_002046	glial fibrillary acidic protein isoform 1
NP_000884	kininogen 1 isoform 2
NP_002639	non-specific serine/threonine protein kinase
NP_000362	transthyretin
NP_000020	angiotensinogen
NP_000057	complement component 8, beta polypeptide
NP_004681	LATS homolog 1
NP_004522	molybdopterin synthase large subunit MOCS2B
NP_005601	retinoblastoma binding protein 4 isoform a
NP_006210	catalytic phosphatidylinositol 3-kinase beta
NP_001966	enolase 2
NP_037398	catenin, alpha 3
NP_055532	zinc finger protein 536
NP_057270	serine (or cysteine) proteinase inhibitor, clade A, member 10
NP_000598	orosomucoid 1
NP_064576	mitochondrial ribosomal protein S22
NP_005680	ATP-binding cassette, sub-family B, member 6
NP_067039	zinc finger protein 71
NP_003277	DNA topoisomerase I
NP_061819	N-acetylneuraminic acid phosphate synthase
NP_073737	axotrophin
NP_001399	cadherin EGF LAG seven-pass G-type receptor 2
NP_116562	serine/threonine kinase 31 isoform b
NP_071407	cadherin-like 23 isoform 1
NP_438171	coronin, actin binding protein, 2A
NP_612121	G6B protein isoform G6b-G
NP_054768	acyl-Coenzyme A dehydrogenase family, member 9
NP_057032	solute carrier family 35, member B3
NP_003600	HIRA interacting protein 3
NP_071394	claspin
NP_683721	phosphatidylinositol glycan anchor biosynthesis, class Q isoform 1
NP_690042	testin isoform 2
NP_056292	transcriptional co-repressor Sin3A
NP_037436	zinc finger, DHHC-type containing 1
NP_060439	IWS1 homolog
NP_065868	hypothetical protein LOC57577
NP_065941	p90 autoantigen
NP_777618	ATPase, Ca++ transporting, ubiquitous isoform c
NP_789847	PDZ and LIM domain 2 isoform 1
NP_065825	mindbomb homolog 1
NP_653266	sestrin 3
NP_066278	macrophage stimulating 1 (hepatocyte growth factor-like)
NP_663327	scavenger receptor class F, member 1 isoform 5
NP_004635	adaptor-related protein complex 3, beta 2 subunit
NP_031394	RAS p21 protein activator 3
NP_942127	ATP citrate lyase isoform 2
NP_065826	hypothetical protein LOC57535

NP_075561	hypothetical protein LOC65250
NP_839943	IQ motif containing GTPase activating protein 3
NP_065931	zinc finger protein 530
NP_009049	triple functional domain (PTPRF interacting)
NP_112582	pseudouridylate synthase 7 homolog (S. cerevisiae)-like
NP_001001583	phosphodiesterase 9A isoform o
NP_203751	FH2 domain containing 1
NP_001003722	GLE1 RNA export mediator homolog isoform 1
NP_000029	adenomatous polyposis coli
NP_055413	suppressor of cytokine signaling 7
NP_065789	kinase D-interacting substrate of 220 kDa
NP_996830	transient receptor potential cation channel, subfamily M, member 3 isoform g
NP_689854	AT rich interactive domain 2 (ARID, RFX-like)
NP_055871	ribosomal RNA processing 1 homolog B
NP_001012300	microspherule protein 1 isoform 2
NP_000177	complement factor H isoform a
NP_001017969	hypothetical protein LOC158358
NP_001724	complement component 1, r subcomponent
NP_056261	transcription factor ELYS
NP_055939	death-inducing-protein
NP_115587	lysyl oxidase-like 4
NP_002854	liver glycogen phosphorylase isoform 1
NP_775864	beta 1,4-N-acetylgalactosaminyltransferase-transferase 3
NP_006176	nuclear mitotic apparatus protein 1
NP_060542	protein BAP28
NP_001029288	eukaryotic translation initiation factor 2B, subunit 4 delta isoform 2
NP_001032221	centrobin, centrosomal BRCA2 interacting protein isoform beta
NP_570856	ATPase, H+ transporting, lysosomal V0 subunit a4
NP_689484	LON peptidase N-terminal domain and ring finger 1
XP_948701	PREDICTED: hypothetical protein
NP_001099041	dehydrogenase/reductase (SDR family) member 7C
NP_038477	bromodomain adjacent to zinc finger domain, 2A
NP_001035197	mutL homolog 3 isoform 1
NP_055333	zinc finger protein 229
NP_001035881	zinc finger protein 596
NP_001092688	RAD51 associated protein 2
XP_944674	PREDICTED: similar to mCG140660
XP_001132038	PREDICTED: hypothetical protein
NP_056073	FK506 binding protein 15, 133kDa
XP_001130382	PREDICTED: similar to Mucin-5AC
XP_001129065	PREDICTED: similar to Opioid growth factor receptor (OGFr) (Zeta-type opioid receptor)
NP_060770	zinc finger protein 83 isoform a
XP_001132875	PREDICTED: hypothetical protein
NP_004534	nebulin isoform 3
NP_689810	fibrous sheath interacting protein 1
NP_115667	SH3-domain GRB2-like (endophilin) interacting protein 1
NP_006303	nuclear receptor co-repressor 2 isoform 1
NP_689561	WD repeat domain 81 isoform 2
NP_036442	kinesin family member 4
NP_004183	acetylserotonin O-methyltransferase-like
NP_000195	complement factor I
NP_004110	fms-related tyrosine kinase 3
NP_659410	DENN/MADD domain containing 5B
NP_001073960	USP6 N-terminal like isoform 2
NP_001073982	carboxypeptidase N, polypeptide 2
NP_758439	exonuclease GOR
NP_002208	inter-alpha (globulin) inhibitor H3

Proteins Down-Regulated in Median-Scorers Compared to High-Scorers

Genbank Accession	Protein Name
NP_002590	phosphodiesterase 2A isoform 1
NP_002821	protein tyrosine phosphatase, non-receptor type 4
NP_002890	retinol binding protein 1, cellular isoform a
NP_113628	transmembrane protein 7
NP_002060	guanine nucleotide binding protein (G protein), alpha inhibiting activity polypeptide 1
NP_940959	RNA binding motif protein 43
NP_055073	synaptonemal complex protein 2
NP_005838	solute carrier family 23 (nucleobase transporters), member 1 isoform a
NP_005086	zinc finger protein 262
NP_000414	keratin 2
NP_001001894	tetratricopeptide repeat domain 3
NP_006581	ubiquitin specific protease 39
NP_002490	neogenin homolog 1
NP_003623	contactin associated protein 1
NP_002920	rhodopsin kinase
NP_000071	nicotinic acetylcholine receptor epsilon polypeptide
NP_005621	solute carrier organic anion transporter family, member 2A1
NP_055269	sestrin 1
NP_068598	FAST kinase domains 5
NP_075066	mitochondrial ribosomal protein L44
NP_116215	solute carrier family 35, member B4
NP_612569	B-cell CLL/lymphoma 11A isoform 3
NP_060478	FEZ family zinc finger 2
NP_004265	A-kinase anchor protein 6
NP_689983	hypothetical protein LOC255119
NP_003161	suppressor of Ty 6 homolog
NP_443732	testis-specific serine kinase 2
NP_005905	minichromosome maintenance complex component 4
NP_940962	lipoma HMGIC fusion partner-like 4
NP_056323	TCDD-inducible poly(ADP-ribose) polymerase
NP_003631	inhibitor of kappa light polypeptide gene enhancer in B-cells, kinase complex-associated protein
NP_036548	RAN binding protein 6
NP_056256	olfactomedin-like 2B
NP_434700	caspase recruitment domain protein 9 isoform 1
NP_003950	huntingtin interacting protein-1-related
NP_065778	zinc finger protein 295 isoform L
NP_777601	scavenger receptor cysteine-rich type 1
NP_981949	adenosine monophosphate deaminase 2 (isoform L) isoform 3
NP_006222	DNA-directed DNA polymerase epsilon
NP_057159	vacuolar protein sorting 36
NP_001026887	cysteine-rich with EGF-like domains 1 isoform 1
NP_001029366	exosome component 9 isoform 1
XP_948609	PREDICTED: hypothetical protein
NP_542789	transmembrane cochlear-expressed protein 2
NP_001036064	v-erb-a erythroblastic leukemia viral oncogene homolog 4 isoform JM-a/CVT-2
NP_056083	DnaJ (Hsp40) homolog, subfamily C, member 13
XP_001126010	PREDICTED: hypothetical protein LOC23514
XP_001126422	PREDICTED: hypothetical protein
XP_948838	PREDICTED: hypothetical protein
NP_001837	alpha 2 type IV collagen
NP_115541	leucine-rich repeats and IQ motif containing 1 isoform 2
